# CGRP Inhibitors and Oxidative Stress Biomarkers in Resistant Migraine: A Real-Life Study with Erenumab, Fremanezumab, and Galcanezumab

**DOI:** 10.3390/jcm10194586

**Published:** 2021-10-04

**Authors:** Ciro De Luca, Filippo Baldacci, Sonia Mazzucchi, Irene Lombardo, Letizia Curto, Martina Ulivi, Lucia Chico, Michele Papa, Gabriele Siciliano, Sara Gori

**Affiliations:** 1 Laboratory of Morphology of Neuronal Network, Department of Public Medicine, University of Campania “Luigi Vanvitelli”, 80138 Napoli, Italy; ciro.deluca@unicampania.it (C.D.L.); michele.papa@unicampania.it (M.P.); 2 Neurology Unit, Department of Clinical and Experimental Medicine, University of Pisa, 56126 Pisa, Italy; mazzucchi.s@gmail.com (S.M.); irene.lombardo@hotmail.com (I.L.); letiziacurto@hotmail.it (L.C.); martinaulivi@gmail.com (M.U.); l.chico@ao-pisa.toscana.it (L.C.); gabriele.siciliano@unipi.it (G.S.); sara-gori@libero.it (S.G.); 3 SYSBIO Centre of Systems Biology ISBE.ITALY, University of Milano-Bicocca, 20126 Milano, Italy

**Keywords:** resistant migraine, CGRP, oxidative stress, biomarkers, medication-overuse headache

## Abstract

Patients with high-frequency resistant migraine and medication-overuse headache are still the main clinical challenge in tertiary headache centers. The approval of targeted antibodies against the calcitonin gene-related peptide (CGRP) and its receptor represents a powerful instrument. In this study, we observed how biological and clinical features of resistant migraineurs responded to erenumab, fremanezumab, or galcanezumab. We found a reduction in advanced oxidation protein products (AOPP) as a biomarker of improved redox state after six months of treatment. We also found that treatment efficacy was precocious and maintained with high individual responder rates. In particular, seven out of ten patients achieved a reduction of 50% from the baseline at three months, which was maintained at six months, while about one out of our patients experienced a 75% reduction in headache frequency from the first month of treatment. The migraine disability assessment (MIDAS) and the associated fatigue, anxiety, and sleep quality also significantly improved. The allodynia symptom dropped from moderate/severe to mild/absent as a sign of central sensitization reduction. Our study confirmed the safety and efficacy of CGRP inhibition in real-life, high-challenging patients. Additional evidence is needed to understand the role of oxidative stress as a migraine biomarker.

## 1. Introduction

Migraine with or without an aura is one of the most frequent and disabling neurological diseases [1,2]. Migraine affects young, prevalently female, patients during their most productive and socially active years of life. The migraine disease expresses highly variable phenotypes considering just the frequency and intensity of the attacks. The pain phase of migraine is usually treated with non-steroidal anti-inflammatory drugs (NSAIDs), triptans, or a combination of analgesics. Prophylactic drugs are also available for patients with more than four days/month of headache to reduce the frequency of the attacks. All these drugs were used for other conditions (e.g., depression, epilepsy, hypertension, spasticity) and found to be effective for migraine treatment [3]. In the last few years, the approval of targeted drugs represented a paradigm shift for migraine, as these molecules designed to block the signaling of the calcitonin gene-related peptide (CGRP), which is thought to be the main culprit for migraine pathogenesis [4,5].

The clinical challenge is still harsh, especially for the chronic migraineurs, which suffer more than 15 days/month of headache and in many cases present both prophylactic failure (resistant or even refractory migraine) and acute medication overuse [6]. The lack of personalized therapy is one of the unmet needs of migraine. Even though comorbidities usually guide the clinician to prefer a prophylactic or acute medication over another, there is still no possibility to predict drug efficacy before its assumption. The trial and error prescriptions that the patient experiences, even when correctly informed by the specialist, are psychologically detrimental [7].

A novel biomarker could both shed some light on the migraine pathophysiology and lead towards precision-medicine targets [8,9,10,11].

Hemodynamic changes related to neurotransmitters concentration, physical ac-tivity, and the associated oxidative stress are important fields of investigation in mi-graine to achieve this goal [12,13].

We previously proposed that the oxidative mitochondrial metabolism could play a role, particularly in chronic migraine with central sensitization [14]. The clinically accessible dosage of plasmatic levels of advanced oxidation protein products (AOPP), ferric-reducing antioxidant power (FRAP), and thiolic groups (-SH) are affordable, though indirect, measurements of the patient’s redox state. They turned out to be significantly different between chronic migraine patients and healthy controls and positively modulated after OnabotulintoxinA (BoNT/A) treatment [14,15]. The exact mechanisms of BoNT/A effectiveness in chronic migraine prophylaxis are not clear; however, lower peripheral plasmatic levels of CGRP have been reported after BoNT/A treatment [16]. However, aside from nociception, CGRP plays a role in oxidative stress, as reported by recent studies [17,18,19].

The objectives of this study were: (i) to examine the levels of the plasmatic oxidative stress biomarkers AOPP, FRAP, and -SH in resistant migraineurs after six months of treatment with monoclonal antibodies against CGRP or its receptor and (ii) to evaluate possible variations of migraine clinical features and comorbidities through validated scales.

## 2. Materials and Methods

### 2.1. Study Population

We carried out a prospective study recruiting consecutive outpatients of the Headache Centre of the University of Pisa, from July 2020 to June 2021, with the enlisted inclusion (IC) and exclusion criteria (EC). IC: (1) adult patient, age ≥ 18 years; (2) patient fulfilling the International Classification of Headache Disorders-3 (ICHD-3) criteria for episodic migraine without or with aura or chronic migraine, and medication-overuse headache (ICHD-3 1.1 or 1.2 or 1.3, and 8.2) [1]; (3) frequency of headache between 8 and 14 days/month for patients with episodic migraine (ICHD-3 1.1 or 1.2); (4) patient fulfilling the European Headache Federation (EHF) consensus on the definition of resistant migraine [20]; (5) absence of migraine preventive treatment for at least 3 months before study inclusion; (6) eligibility for erenumab, fremanezumab, and galcanezumab. EC: (1) comorbid medical disorders and treatments for chronic systemic diseases; (2) pregnancy or breastfeeding during the study.

All patients were not enrolled in previous clinical studies on migraine and were not previously treated with monoclonal antibodies against CGRP. This study was performed in accordance with the Declaration of Helsinki, and it was approved by the local ethics committee (Comitato Etico Area Vasta Nord Ovest—Sezione Autonoma del Comitato Etico Regionale per la Sperimentazione Clinica—Via Roma 67, 56126, Pisa, Italy) with approval code ID_14518. All subjects involved provided written, informed consent before their inclusion.

### 2.2. Clinical Assessment

Patients underwent four visits: baseline assessment (T0) and 1-month (T1), 3-months (T2), and 6-months (T3) follow-up visits. At the end of each visit (T0, T1, T2, and T3), a blood sample was collected for biochemical analysis. Each visit was performed before the administration of the drug. To resolve the different periodicity of the treatment, a standard month of 30 days was considered with a -4 days window to schedule the next visit of patients receiving galcanezumab and fremanezumab and a -2 days window for patients treated with erenumab.

Furthermore, clinical features of migraine were measured through an interview and patients’ self-reported diary as well as the following scales: Migraine Disability Assessment (MIDAS) for the assessment of migraine-related disability (T0, T2, T3), Allodynia Symptoms Checklist 12 (ASC-12) for a specific measure of ictal allodynia (T0, T3), the Fatigue Severity Scale (FSS) to evaluate migraine-associated fatigue (T0, T3), and the Generalized Anxiety disorder (GAD-7) and Patient Health Questionnaire (PHQ-9), to evaluate anxiety and symptoms of mood disorders (T0, T3). Sleep quality was determined (T0, T3) through the Pittsburgh Sleep Quality Index (PSQI). The migraine frequency was evaluated on a single month for T1 and over the previous two months and three months for T2 and T3, respectively.

### 2.3. Biochemical Analysis

Blood samples were collected during the T0, T1, T2 and T3 visits. All the venous samples were collected early in the morning (around 9 a.m.), after a light breakfast. Blood sample vials were centrifuged within 2 h from the collection (2500 g for 10 min −4 °C) for plasma separation and processed afterward or stored at −80 °C and analyzed within 1 month. Plasmatic levels of AOPP, FRAP, and -SH were measured as previously described [14]. The biochemical analysis was performed at the laboratory of the Neurology Unit.

AOPP are indicators of oxidative damage, particularly to proteins, including bioproducts of the myeloperoxidase enzyme reacting with plasma proteins. Data were shown as nmol/mL of chloramine equivalents (CE).

The FRAP measurement estimates the non-enzymatic antioxidant properties of plasma. The data were expressed as mmol/L.

The measure of sulfhydryl groups (-SH), also known as thiol groups, expressed the property shared by some plasma molecules, such as glutathione (GSH), to oppose the propagation of oxidative processes and enzymatically revert the oxidation. Data were expressed as mol/L.

### 2.4. Statistical Analysis

Quantitative variables were not normally distributed (Shapiro–Wilk test). The continuous variables were expressed as medians and interquartile ranges (IQR). The categorical variables were expressed as frequency percentages. The comparison between non-parametric quantitative variables was performed employing the Wilcoxon test to compare the difference at T0, T1, T2, and T3 of the quantitative variables within the migraine population. The statistical significance threshold was set at *p* > 0.05. A correction for multiple comparisons with Bonferroni was applied for biochemical analysis (*p* = 0.016). The comparison between categorical dichotomous variables was performed by the chi-square test with continuity correction (Yates or Fisher exact test when necessary). SPSS version 24.0 for Windows was used for statistical analyses

## 3. Results

The patients enrolled in the study were 42, of whom 33 (78.6%) were females, and 9 (21.4%) were males; their ages comprised between 27 and 74 years old, with a median of 52 (14.75). All the patients were diagnosed, according to the ICHD-3 as migraine without aura, and 4 patients (9.5%) also received the migraine-with-aura diagnosis. High-frequency episodic migraine was assessed in 6 patients (14.3%), while the remaining 36 (85.7%) were diagnosed with chronic migraine. All chronc migraine patients fulfilled the criteria for medication-overuse headache (MOH).

The years of illness were between 10 and 57 years with a median of 32.5 (IQR 14.5).

The patients were treated as follows: 23 received erenumab 70 mg; galcanezumab was prescribed to 17 patients (240 mg loading dose, 120 mg monthly); 2 patients were enrolled with fremanezumab 225 mg.

Two patients experienced slight side effects (pain at the site of injection), one with erenumab and one with galcanezumab, while none discontinued the prescribed drug.

All our patients were resistant to four of the recommended classes of prophylactic drugs considering antiepileptics, tricyclic antidepressants, beta-blockers, angiotensin-receptor blockers (ARBs), and calcium-channel blockers. Two-thirds (30 patients) were also resistant to selective serotonin reuptake inhibitors (SSRIs) or serotonin-norepinephrine reuptake inhibitors (SNRIs). Eventually, 25 patients underwent at least 3 cycles of BoNT/A administration.

The headache frequency (Table 1), expressed in days/month, for all the patients underwent a significant decrease considering the T0—T1 comparison (T0: 22.0, IQR 14; T1: 12.0, IQR 12; *p* < 0.001).

The analysis of the T2 and T3 time points revealed a stable improvement in the headache frequency. In particular, for the 33 patients, we observed at T2 (3 months from the baseline) a significantly lower median of 8 days/month (IQR 11.5), compared to the T0 (*p* < 0.001). The same can be stated about the 22 patients at T3 (9.0, IQR 8, *p* = 0.003).

After three months of treatment, 61.3% of patients (19 out of 31) initially diagnosed with MOH and chronic migraine suspended the overuse. The result was even more evident at T2 with 70% of patients (14 out of 20) that achieved MOH cessation.

The MIDAS score (Table 1) showed an improvement in the quality of life with the reduction in the migraine-related disability (81, IQR 69) both at T2 (23.5, IQR 31.25; *p* < 0.001) and T3 (14, IQR 17.75; *p* < 0.001).

Another endpoint we considered was the individual responder rate, for reduction in the headache frequency higher than 50% or 75% compared to the baseline.

Compared to T0, 51.2% of patients achieved a 50% reduction in their headache frequency after 1 month of treatment, while 69.7% achieved this endpoint at T2, and approximately the same percentage maintained it at 6 months (68.2%).

Moreover, a reduction of 75% in the baseline headache frequency was observed consistently at all time points, specifically: 24.4% of patients after 1 month, 36.4% at T2, and 27.3% at T3.

**Figure 1:**
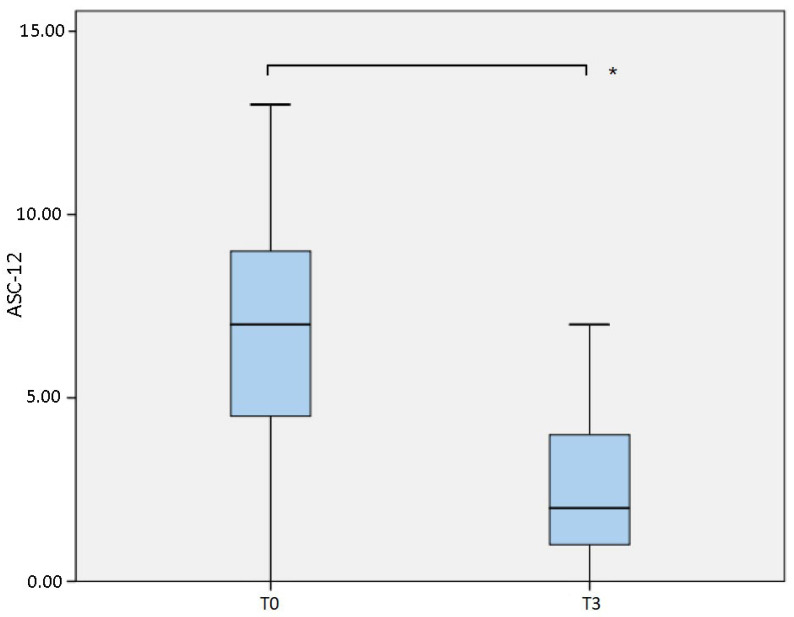
Comparison of Allodynia Symptoms Checklist 12 (ASC-12) measured at baseline (T0) and after 6 months (T3). * *p* = 0.001.

Allodynia was significantly reduced as indicated by the ASC-12 evaluation (Table 1, Figure 1), which showed a median value of 7 (IQR 5) at T0 and which dropped to 2 (IQR 3; *p* = 0.001) at T3.

Furthermore, the associated fatigue was significantly reduced comparing T0 and T3 (T0: 45, IQR 18; T3: 27, IQR 20.25; *p* = 0.038).

Considering the anxiety symptoms, the GAD-7 score indicated a significant decrease from a median of 9 (IQR 9) at T0 to 5 (IQR 2.5) after 6 months of therapy (*p* = 0.002), while the mood impairment, assessed through the PHQ-9, showed no significant differences (Table 1). 

The PSQI demonstrated a significant reduction from a median of 11 (IQR 11) to 7 (IQR 6.5) comparing the baseline to T3 (*p* = 0.004); however, it did not reach the cutoff of <5 for a good quality of sleep (Table 1).

**Figure 2:**
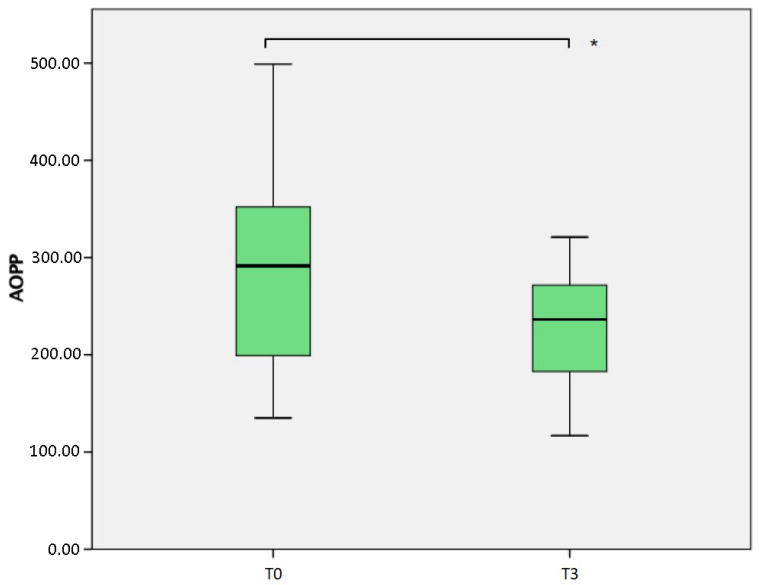
Comparison of Advanced Oxidation Protein Products (AOPP) measured at baseline (T0) and after 6 months (T3). * *p* = 0.004.

A significant reduction in the advanced oxidation products AOPP (Table 2, Figure 2) was measured (*p* = 0.004), comparing the samples obtained at T0 (median 291.4 nmol/L, IQR 154.25) and T3 (median 236.2 nmol/L, IQR 111.2). The result was obtained considering 37 patients at baseline and 11 patients at T3. If the comparison is conducted considering the same 11 patients at both T0 (median 336.0 nmol/L, IQR 91.3) and T3, the difference is even more evident (*p* = 0.004).

The values indicating the antioxidant power of the plasma showed no significant differences with the baseline and both the FRAP and -SH (Table 2). The latter exhibited an increasing trend at T1 (median 0.271μmol/L, IQR 0.062), T2 (0.276 μmol/L, IQR 0.071), and T3 (median 0.272 μmol/L, IQR 0.061) compared to T0 (median 0.235 μmol/L, IQR 0.0815), however without reaching statistical significance.

## 4. Discussion

The population of the study was selected to express a severe phenotype of migraine (either high-frequency episodic or chronic migraine), with all chronic migraine patients being diagnosed with MOH and all patients fulfilling the EHF definition of resistant migraine.

Nonetheless, the CGRP system inhibition (erenumab, fremanezumab, galcanezumab) managed to improve the clinical features of migraine. Particularly significant was the related disability measured with MIDAS that, at baseline, had a median value of 81, which was far above the cutoff of 21 for “severe disability” and which dropped to a median value of 14 (*p* < 0.001) at six months in the range of “moderate disability.” The reduction in the MIDAS score was evident also after three months, even remaining in the severe disability range (median value = 23.5).

The treatments improved migraine symptoms, particularly allodynia, which ameliorated from moderate/severe to mild/absent after six months (*p* = 0.001). Considering allodynia as a measure of central sensitization, this result can be interpreted as the positive interference of the therapy on the trigeminovascular system [21] either directly or as a consequence of the clinical improvement. In chronic migraine, the prolonged painful stimulation could induce sensitization, particularly in the caudal trigeminal nucleus and in the trigeminothalamic cortical circuitry, clinically manifesting as allodynia [22].

The clinical improvement was indeed remarkable, with more than half of the patients achieving an individual reduction of 50% in headache frequency after one month, and with almost 7 out of 10 patients experiencing the same improvement after three and six months. The result was coupled with a 61.3% and 70% reduction in patients with a MOH diagnosis at T2 and T3, respectively.

Furthermore, a significant percentage of the patients (24.4% at T1, 36.4% at T2, and 27.3% at T3) reported an individual reduction of 75% in their headache frequency.

These data are corroborating the efficacy of the anti-CGRP treatment even in a severe migraine phenotype.

The associated fatigue, measured through the FSS, consistently showed a significant reduction, as well as the anxiety symptoms (GAD-7), demonstrating an overall improvement in the associated comorbidities, together with the migraine disease itself. The amelioration of sleep quality, on the other hand, even if statistically significant was not enough to reach a good quality of sleep.

This parameter is important, as recently demonstrated during the pandemic when both physical activity levels and sleep quality dropped in migraine patients [23]. The link between oxidative stress, physical activity, and sleep quality could somehow reside in the CGRP system [12,13,23].

The safety of the antibodies’ anti-CGRP, or the associated receptor, was confirmed in this real-life study, considering the minor side effects that never led to drug discontinuation.

The results of this study are in line with previous observations of increased oxidative stress in migraine patients [14,15,17,24,25]. The oxidative state of plasma proteins and the antioxidant reservoir were measured through the biomarkers AOPP, FRAP, and -SH. In our previous investigation, we found that chronic migraine with medication overuse reduced the antioxidant power of plasma [14]. This evidence was subsequently confirmed, together with the increase in AOPP, as a marker of advanced oxidation [15]. Moreover, in the aforementioned study, we investigated the role of BoNT/A considering both clinical and oxidative stress parameters.

We hypothesized that the complex action of the toxin, among other features, chronically blocked the release of CGRP, reducing peripheral sensitization secondary to “neurogenic” inflammation and consequently the oxidative stress [15]. The direct interference with the CGRP system aids the validation of our previous experimental data and adds more information about migraine pathophysiology biomarkers. 

We confirmed a significant reduction in AOPP (*p* = 0.004) with chronic inhibition of the CGRP system, after six months of treatment. We did not demonstrate an increase in FRAP and -SH, as previously reported after BoNT/A treatment. This study suggests that the increase in antioxidant power of the patients’ plasma could be unrelated to the disease severity and at least partly dependent on the chosen treatment.

Studying the biomarkers, the tonic blockage of CGRP signaling did not affect the reduction in the oxidation products, which was also observed with BoNT/A treatment [15]. However, we failed to demonstrate the increase in the antioxidant power of plasma, found with the BoNT/A therapy, either for the different mechanism of action or for the small population size.

Indeed, the CGRP has been involved in antioxidant activation, both in vivo and in vitro, other than in nociception [18,19,24,25,26]. In this scenario, we were not able to define which role is played exclusively by the CGRP-signaling blockage. However, we can speculate that even if both anti-CGRP antibodies and BoNT/A toxin treatments are effective in fairly similar populations, the antibodies failed to improve the antioxidant power of plasma, probably due to the blockage of CGRP antioxidant properties.

This evidence suggests that the CGRP system inhibition is indeed important for both enzymatic and non-enzymatic antioxidant power. However, the concentration of oxidation products was reduced, probably due to the improvement of migraine frequency or as a consequence of medication overuse discontinuation, as we previously suggested considering the BoNT/A treatment [15]. The CGRP system blockage also provides the same result, which could be independent of the chosen treatment, further validating the aforementioned hypothesis.. Our study has some limitations. First of all, the sample is relatively small, impeding sub-population analyses, as episodic versus chronic migraine, triptan versus NSAIDs overuser, or according to the administered antibody, to determine, for instance, if direct CGRP inhibition or its receptor could have different effects on oxidative stress.

Other comparative studies with similar populations are mandatory to further validate our hypotheses.

## 5. Conclusions

This study confirmed the safety and efficacy of the antibodies’ anti-CGRP and its receptor in real-life, with patients characterized by a high-frequency, pharmacologically resistant migraine. The clinical improvement was also accompanied by a reduction in the oxidative stress biomarker, paving the way for the further understanding of migraine pathophysiology.

Additional studies will be necessary to understand the role of oxidative stress as a disease biomarker and how different treatments could affect the advanced oxidation products or the enzymatic and non-enzymatic antioxidant power of patients’ plasma.

## Figures and Tables

**Table 1:** Clinical features comparison at different time points. Values are expressed as median (IQR) and the number of patients (*n*).

	T0	T1	T2	T3	*p* (Wilcoxon Test)
Frequency (days/month)	22.0 (14) *n* = 42	12.0 (12) *n* = 41	8.0 (11.5) *n* = 33	9.0 (8) *n* = 22	T0–T1: *p* < 0.001 *** T0–T2: *p* < 0.001 *** T0–T3: *p* = 0.003 **
MIDAS ^1^	81 (69) *n* = 42	-	23.5 (31.25) *n* = 32	14 (17.75) *n* = 22	T0–T2: *p* < 0.001 *** T0–T3: *p* < 0.001 ***
FSS ^2^	45 (18) *n* = 39			27 (20.25) *n* = 20	*p* = 0.038 *
ASC-12 ^3^	7 (5) *n* = 39			2 (3) *n* = 22	*p* = 0.001 **
GAD-7 ^4^	9 (9) *n* = 39			5 (2.5) *n* = 21	*p* = 0.002 **
PHQ-9 ^5^	8 (8) *n* = 39			5.5 (7) *n* = 22	*p* = 0.393
PSQ I ^6^	11 (11) *n* = 39			7 (6.5) *n* = 20	*p* = 0.004 **

^1^ Migraine Disability Assessment Scale; ^2^ Fatigue Severity Scale; ^3^ Allodynia Symptom Checklist 12; ^4^ Generalized Anxiety Disorder 7; ^5^ Patient Health Questionnaire 9; ^6^ Pittsburgh Sleep Quality Index; (*** *p* < 0.001; ** *p* < 0.01; * *p* < 0.05).

**Table 2:** Biochemical analysis of redox properties of patients’ plasma at different time points. Values are expressed as median (IQR) and the number of patients (*n*).

	T0	T1	T2	T3	*p* (Wilcoxon Test)
AOPP ^1^	291.4 (154.25) *n* = 37	263.2 (139.5) *n* = 19	299.0 (166.3) *n* = 15	236.2 (111.2) *n* = 11	T0–T1: *p* = 0.306 T0–T2: *p* = 0.272 T0–T3: *p* = 0.004 *
FRAP ^2^	0.603 (0.0815) *n* = 37	0.616 (0.062) *n* =19	0.561 (0.071) *n* = 15	0.584 (0.061) *n* = 11	T0–T1: *p* = 0.085 T0–T2: *p* = 0.041 T0–T3: *p* = 0.859
-SH ^3^	0.235 (0.081) *n* = 37	0.271 (0.062) *n* =19	0.276 (0.071) *n* = 15	0.272 (0.061) *n* = 11	T0–T1: *p* = 0.603 T0–T2: *p* = 0.334 T0–T3: *p* = 0.110

^1^ Advanced Oxidation Protein Products; ^2^ Ferric-Reducing Antioxidant Power; ^3^ Thiolic Groups; (* *p* < 0.01).

## Data Availability

The data presented in this study are available on request.
